# Emotional eating as a risk factor for body image and life satisfaction

**DOI:** 10.1192/j.eurpsy.2021.1221

**Published:** 2021-08-13

**Authors:** E. Fyodorova, G. Arina, V. Nikolaeva

**Affiliations:** Psychology, Lomonosov Moscow State University, Moscow, Russian Federation

**Keywords:** body image, satisfaction with life, emotional eating

## Abstract

**Introduction:**

Previous studies have shown that emotional eating is associated with binge eating disorder, body image disturbances and depression.

**Objectives:**

In this study we wanted to find out if there is a relationship between emotional eating and body image and life satisfaction in non-clinical sample.

**Methods:**

The study involved 182 normal participants (153 Female, 29 Male, mean age 22,6 ± 7,3), which were recruited in Moscow, Russia. Emotional eating was measured by the opposite pole of Eating for Physical Rather Than Emotional Reasons subscale of Intuitive Eating Scale-2 (IES-2), body image was measured by Multidimensional Body-Self Relations Questionnaire (MBSRQ), Satisfaction with Life Scale (SWLS) was used to measure the corresponding construct. Correlation analysis was performed in IBM SPSS Statistics 22.0.

**Results:**

Emotional eating was associated with the following MBSRQ subscales: lower appearance evaluation (-0,431, p<0,0001), lower body areas satisfaction (-0,335, p<0,0001), as well as lower fitness evaluation (-0,208, p=0,005) and lower health evaluation (-0,182, p=0,014), but higher overweight preoccupation (0,279, p=0,0001) and overestimation of body weight (0,362, p<0,0001). It was also connected to lower satisfaction with life (-0,195, p=0,008).

**Conclusions:**

The results of the study allow us to conclude that emotional eating may pose risks to psychological health of a normal individual. It was shown that emotional eating is connected to negative evaluation of one`s body appearance, fitness and health state, weight and shape concerns, and even to the lower level of satisfaction with one’s life.

